# Genetically-reduced serum ACE activity might be a causal risk factor for obstructive sleep apnea syndrome: A meta-analysis

**DOI:** 10.1038/srep15267

**Published:** 2015-10-21

**Authors:** Lan He, Bin Wang, Wei-Ya Lang, Jing Xue, Da-Long Zhao, Guo-Feng Li, Li-Hong Zheng, Hong-Ming Pan

**Affiliations:** 1 Basic Medical Science College, Qiqihar Medical University, Qiqihar, Heilongjiang, China

## Abstract

We meta-analytically summarized the associations of angiotensin converting enzyme (*ACE*) gene insertion/deletion (I/D) polymorphism with ACE activity and obstructive sleep apnea syndrome (OSAS) to see whether ACE activity is causally associated with OSAS. Literature search and data abstraction were done in duplicate. Sixteen articles including 2060 OSAS patients and 1878 controls were summarized. Overall, no significance was observed for the association of I/D polymorphism with OSAS, whereas carriers of II genotype (weighted mean difference or WMD, 95% confidence interval or CI, P: −11.976, −17.168 to −6.783, <0.001) or I allele (−9.842, −14.766 to −4.918, <0.001) had a lower level of serum ACE activity compared with DD genotype carriers, respectively. In subgroup analyses, carriers of II genotype were 3.806 times more likely to develop OSAS (95% CI, P: 1.865 to 7.765, <0.001) in OSAS patients with hypertension, without heterogeneity. Mendelian randomization analysis indicated there was 37.4% (95% CI: 1.115 to 3.142) and 32.4% (1.106 to 2.845) increased risk of OSAS by a reduction of 1 U/L in ACE activity for the II genotype and I allele carriers versus DD genotype carriers, respectively. There was no observable publication bias. Collectively, genetically-reduced serum ACE activity might be a causal risk factor for OSAS.

As the most common category of sleep apnea, obstructive sleep apnea syndrome (OSAS) is estimated to affect 4% to 5% of the general population^1^,^2^. OSAS is characterized by recurrent episodes of upper airway obstruction for 10 seconds or more during sleep. There are several established risk factors for OSAS, such as obesity, excessive relaxation of the throat muscles and abnormal structure of the airways^3^,^4^. The symptoms of OSAS are more common in family members affected with this disorder, and it occurs two to three times more often in males than in females^5^,^6^. Currently, the development of OSAS is believed to, at least in part, be genetically determined with a heritability of 0.35^1^, and the gene encoding angiotensin converting enzyme (*ACE*) is recognized as a promising candidate in susceptibility to OSAS.

ACE is involved in catalyzing the conversion of angiotensin I into a biologically active peptide angiotensin II, a potent vasopressor and aldosterone-stimulator that regulates blood pressure and fluid-electrolyte balance^7^. An early study by Rohatgi *et al.* reported that OSAS patients had lower ACE activity than healthy controls^8^, arguing against the finding of subsequent study by Barcelo *et al.*, who reported that ACE activity was significantly higher in OSAS patients than in healthy controls^9^. At present, the relation between ACE activity and OSAS susceptibility remains an open question. Converging studies suggested that serum ACE activity was controlled by an insertion/deletion (I/D) polymorphism of a 287-bp Alu element in the intron 16 of *ACE* gene, with the D/D, I/D and I/I genotypes respectively paralleling the high, middle and low levels of ACE activity^10^,^11^. In view of these observations, we were inspired to see whether there was a causal association between serum ACE activity and OSAS susceptibility with the aid of *ACE* gene I/D polymorphism as an instrument variable under the assumptions of Mendelian randomization by a meta-analysis in accordance with the guidelines of Preferred Reporting Items for Systematic Reviews and Meta-Analyses (PRISMA) (Please see the Supplementary PRISMA 2009 Checklist, downloaded from http://www.prisma-statement.org/statement).

## Methods

### Literature search

Four public electronic datasets, PubMed, Web of Science, Wanfang (http://www.wanfangdata.com.cn/, Chinese) and CNKI (www.cnki.net/, Chinese) were reviewed to search potential articles that tested the associations of *ACE* gene I/D polymorphism or plasma/serum ACE activity with OSAS susceptibility. The key words were “angiotensin converting enzyme” or “angiotensin I converting enzyme” or “ACE”, or “ACE1” in the ABSTRACT, along with “sleep apnea” or “sleep apnoea” or “sleep disorder” or “OSAS” or “OSAHS” in the TITLE. The last search was updated on April 4, 2015. Only articles published in English or Chinese language were considered in this study. In order to avoid possible missing hits, the reference lists of major original articles and reviews were checked manually.

### Selection criteria

Articles were qualified if they tested the associations of *ACE* gene I/D polymorphism or plasma/serum ACE level/activity with OSAS susceptibility; if they involved both OSAS patients and healthy controls; if they provided the genotype or allele counts of *ACE* gene I/D polymorphism between the two groups or the mean plasma/serum ACE activity across I/D genotypes. Article selection process was independently completed by two authors (Lan He and Bin Wang) and there was no disagreement.

### Data abstraction

The following data were abstracted from each qualified article by two authors (Lan He and Bin Wang) independently based on a predetermined protocol: the first author’s last name, year of publication, race, study design, source of healthy controls, diagnostic criteria for OSAS and apnea hypopnea index (AHI) cutoff, as well as sample size, age, percentage of male gender, body mass index (BMI), smoking, drinking, AHI cutoff, Epworth sleepiness score, hypertension and diabetes mellitus between OSAS patients and healthy controls.

### Mendelian randomization

Mendelian randomization is emerging as a novel epidemiologic study design that utilizes measured variation in genes of known biology function to examine the causal impact of a modifiable exposure on disease susceptibility in genetic association studies^12^. Importantly, this design can control for reverse causation and confounding which otherwise obsess observational data. To infer a causal relation between ACE activity and OSAS, we firstly suppose that the mutant II genotype increases OSAS susceptibility relative to the wild DD genotype as measured by OR_II vs. DD_. Secondly, we suppose that the mean difference of ACE activity between II genotype and DD genotype is expressed as ΔP. Thirdly, under the assumptions of Mendelian randomization, OR_GG vs. gg_^1/ΔP^ is regarded as an unbiased and unconfounded estimate of the OR of OSAS for a unit change in ACE activity^12^,^13^.

### Statistical analysis

Inconsistency index, *I*^2^, quantifies the magnitude of heterogeneity as a percentile, with a higher value paralleling a higher probability of heterogeneity. In fact, *I*^2^ is a derivative of the Q statistic and it measures the proportion of variability that is due to heterogeneity rather than sampling error. Generally, the cutoff of *I*^2^ is set at 50% to define statistically significant heterogeneity^14^. Associations of *ACE* gene I/D polymorphism and ACE activity with OSAS susceptibility were respectively quantified as odds ratio (OR) and weighted mean difference (WMD), with 95% confidence interval (95% CI) by the DerSimonian and Laird random-effects model^15^.

Subgroup analyses were conducted to see whether differences in race (mainly Asian and White), language of publication (English and Chinese), study design (retrospective and prospective), source of controls (hospital-based and population-based), obesity (BMI ≥ 30 kg/m^2^ and BMI < 30 kg/m^2^), hypertension status (with and without hypertension) and sample size (≥300 subjects and <300 subjects) can explain heterogeneity. Moreover, meta-regression analyses were conducted on continuous covariates including age, gender, BMI, smoking, drinking to seek possible explanations for heterogeneity.

Publication bias was measured by Begg’s funnel plots and Egger’s regression tests, as well as by the trim-and-fill test, which can calculate the number of estimated missing studies to compensate for publication bias. Significant publication bias was statistically judged by the P value of Egger’s regression test at a significance level of 10%.

The above statistical analyses were managed by STATA software version 12.0 for Windows (College Station, TX: StataCorp LP).

## Results

A comprehensive review of public electronic datasets produced a total of 179 eligible articles (62 articles in English and 117 articles in Chinese) that tested the associations of *ACE* gene I/D polymorphism and ACE activity with OSAS susceptibility. Only 16 articles (9 articles in English and 7 articles in Chinese) including 2060 OSAS patients and 1878 controls were left for final analysis after applying eligibility criteria^9^,^16^,^17^,^18^,^19^,^20^,^21^,^22^,^23^,^24^,^25^,^26^,^27^,^28^,^29^,^30^. The baseline characteristics of all qualified studies are summarized in Table 1.

**Figure 1:**
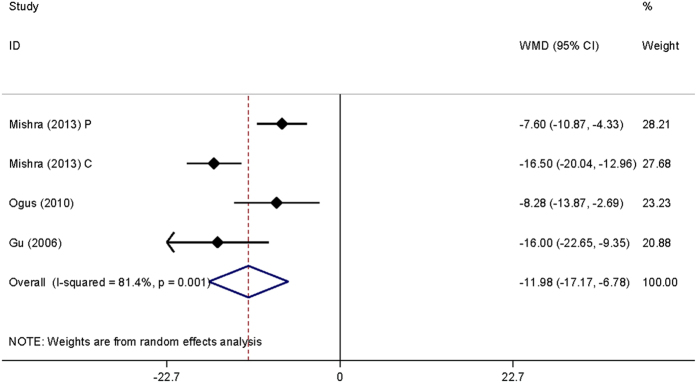
Forest plot of serum ACE activity for the comparison of *ACE* gene II genotype with DD genotype.

When all study subjects were brought together, no significance was observed for the association of *ACE* gene I/D polymorphism with OSAS susceptibility under allelic, homozygous genotypic and dominant models, and heterogeneity was significant (Table 2). However, carriers of II genotype (WMD, 95% CI, P: −11.976, −17.168 to −6.783, <0.001) or I allele (−9.842, −14.766 to −4.918, <0.001) had a lower level of serum ACE activity when compared with DD genotype carriers, with significant heterogeneity (Fig. 1).

**Figure 2:**
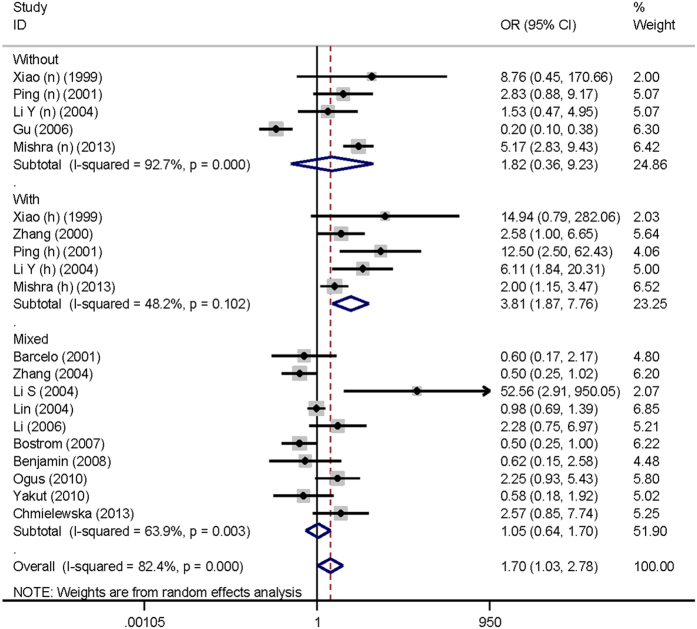
Forest plot of OSAS susceptibility conferred by the comparison of *ACE* gene II genotype with DD genotype by hypertension status.

For the association of *ACE* gene I/D polymorphism with OSAS, further subgroup analyses were carried out to seek possible sources of heterogeneity. As shown in Table 2, grouping studies by race, language of publication, study design, source of controls and sample size failed to find any statistical significance, and no obvious improvement in heterogeneity was noticed. In subgroup analysis by obesity (BMI at a cutoff of 30 kg/m^2^ in OSAS patients), significance was detected in OSAS patients with BMI of less than 30 kg/m^2^ under only allelic model (OR, 95% CI, P: 1.708, 1.047–2.785, 0.032), with evident heterogeneity (*I*^2^ = 79.8%). In addition, four of 16 qualified articles that reported genotype data by hypertension status were analyzed separately. In OSAS patients complicated with hypertension, the significant associations of *ACE* gene I/D polymorphism with OSAS susceptibility were identified under three genetic models. For example, carriers of II genotype were 3.806 times more likely to develop OSAS (OR, 95% CI, P: 3.806, 1.865 to 7.765, <0.001), and there was no indication of significant heterogeneity (*I*^2^ = 48.2%) (Fig. 2).

**Figure 3:**
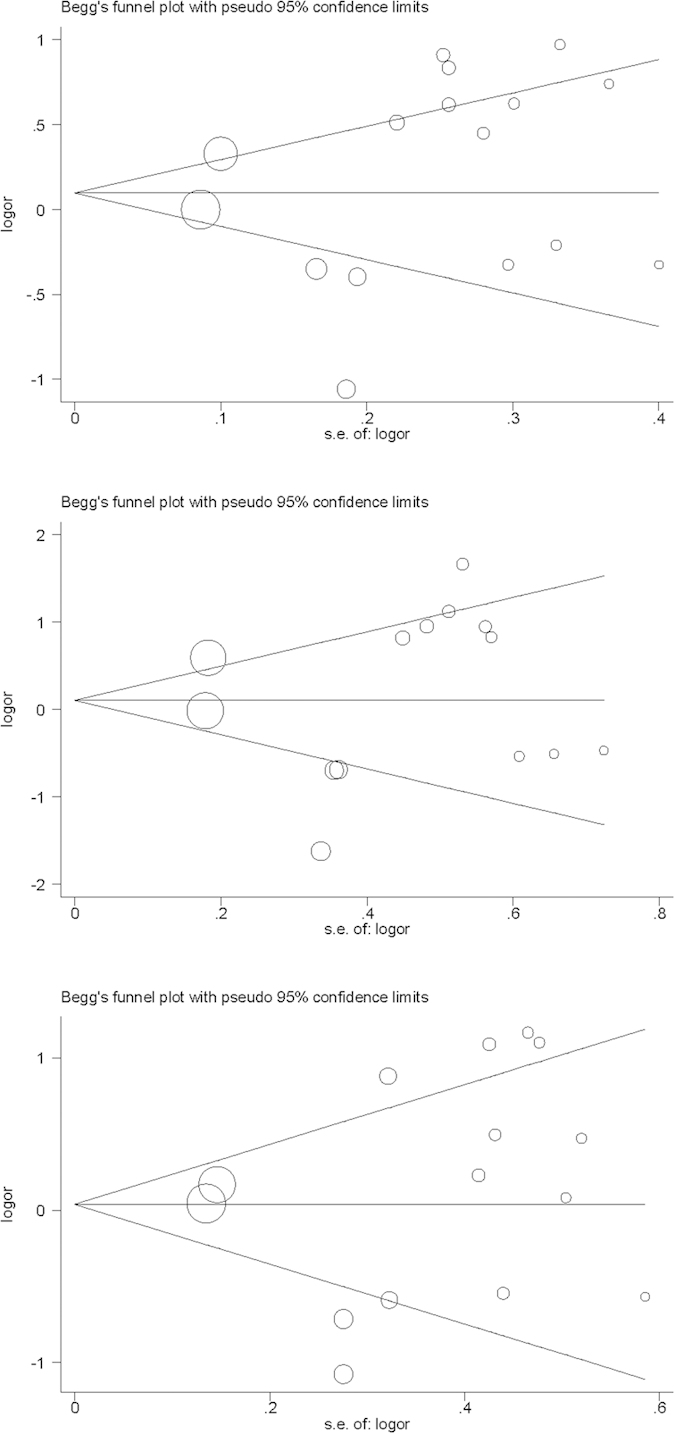
Begg’s funnel plots for the I allele versus D allele (the upper), II genotype versus DD genotype (the middle), and II plus ID genotypes versus DD genotype (the lower).

Further, meta-regression analyses modeling age, gender, BMI, smoking and drinking showed no statistical significance for the association between *ACE* gene I/D polymorphism and OSAS susceptibility. Echoing from Begg’s funnel plots and Egger’s regression tests, there was no suggestive publication bias (Fig. 3). As suggested by the trim-and-fill method, 3 missing studies were required for *ACE* gene I allele in association with OSAS susceptibility, with the trim-and-fill-adjusted OR of 1.029 (95% CI, P: 0.791 to 1.338, 0.833).

On the basis of above estimates, implementation of Mendelian randomization identified 37.4% (OR, 95% CI: 1.374, 1.115 to 3.142) and 32.4% (1.324, 1.106 to 2.845) increased risk of developing OSAS by a reduction of 1 U/L in serum ACE activity for the II genotype and I allele carriers versus the DD genotype carriers, respectively.

## Discussion

Polling previous studies on the ACE-OSAS relation, our findings demonstrate that *ACE* gene I/D polymorphism can predict the risk for OSAS patients complicated with hypertension, and more importantly, genetically-reduced serum ACE activity might be a causal risk factor for OSAS by Mendelian randomization technique. To the best of our knowledge, this meta-analysis represents the first to test the causal contribution of ACE activity in the pathogenesis of OSAS.

Among the promising candidate genes identified so far in susceptibility to OSAS, *ACE* gene ranks high on the list. In spite of many candidate association studies conducted in different ethnic groups, data in medical literature are inconsistent and even contradictory, and biological implication from statistical inference remains elusive. Lately, two meta-analyses found no relationship between *ACE* gene I/D polymorphism and OSAS susceptibility^31^,^32^. Moreover, there is also ongoing discordance in terms of ACE activity and OSAS^8^,^9^,^23^,^27^. A possible explanation for these divergent findings may be due to clinical or methodological heterogeneity across studies, such as the coexistence of hypertension. Another possibility might result from confounding and reverse causation inherited in observational data^33^. Indeed, inferring causality from genetic association studies is problematic because it is difficult to disentangle causation from an association, especially in the presence of confounding^34^. Fortunately, Mendelian randomization provides an alternative way to deal with the problems of observational studies^35^. This technique is alike to a randomized trial where randomization to genotypes takes place at the time of gamete formation. With these in mind, we therefore sought to update previous meta-analyses to seek potential sources of heterogeneity and further introduce the concept of Mendelian randomization to test whether there is a causal relation between ACE activity and OSAS susceptibility.

In agreement with the previous two meta-analyses^31^,^32^, our overall analyses repeated the overall neutral association between *ACE* gene I/D polymorphism and OSAS susceptibility. To produce more information, we subsequently conducted a wide range of subgroup analyses to seek possible sources of heterogeneity resulting from clinical and methodological aspects, and interestingly a positive signal emerged after restricting data to OSAS patients complicated with hypertension and normotensive healthy controls. It is well recognized that OSAS is a risk factor for the development of hypertension and approximately half of OSAS patients have hypertension^36^,^37^. Elucidating the pathophysiological mechanisms of *ACE* gene underlying OSAS susceptibility in connection with hypertension is beyond the capability of this meta-analysis, it is rational to speculate that the predictive role of *ACE* genetic alterations is manifested in the coexistence of hypertension.

The most noteworthy finding of this study is that our Mendelian randomization analysis demonstrated that genetically-reduced serum ACE activity might be a causal risk factor for OSAS with the aid of *ACE* gene I/D polymorphism as an instrument variable. Factually, the selection of I/D polymorphism as a surrogate to infer causality between ACE activity and OSAS is biologically robust as this polymorphism was reported to interpret half the variance of serum ACE activity^10^. As expected from our results, the II genotype was associated with a significant lower level of ACE activity as compared with the DD genotype, in agreement with the findings of most studies^23^,^27^. In this meta-analysis, we for the first time put forward that a unit reduction of serum ACE activity can lead to 30% increased risk of predisposing OSAS. However, given the complex pathogenesis of OSAS and its underlying relation with hypertension, more and more large studies are warranted to validate this significant finding.

There are several research limitations for this meta-analysis. The conclusion of this study was based on 16 independent articles and 3938 subjects, which might not be sufficient enough to quantify the effect estimate reliably. Only one polymorphism in *ACE* gene was selected as a surrogate marker, and it is encouraged to bring other functional polymorphisms within or flanking *ACE* gene together to examine their joint impact on OSAS susceptibility and ACE activity. Moreover, as with a majority of meta-analyses, heterogeneity is a disturbing issue for both overall and subgroup analyses, and seeking other sources of between-study heterogeneity still remains a challengeable task. Further, all retrieved articles were published in English or Chinese language, leading to a possibility of selection bias. However, as viewed from Begg’s funnel plots and Egger’s tests, there was no indicative of publication bias, substantiating the robustness of our findings.

In conclusion, through a well-designed Mendelian randomization meta-analysis, our findings demonstrate that *ACE* gene I/D polymorphism can predict the risk for OSAS complicated with hypertension, and more importantly, genetically-reduced serum ACE activity might be a causal risk factor for OSAS. Nevertheless, the results of this meta-analysis need further replication in other ethnic groups, and *in-vivo* and *in-vitro* functional studies are also required to unravel potential molecule mechanisms underlying the associations of *ACE* genetic defects and its serum activity with OSAS susceptibility.

## Additional Information

**How to cite this article**: He, L. *et al.* Genetically-reduced serum ACE activity might be a causal risk factor for obstructive sleep apnea syndrome: A meta-analysis. *Sci. Rep.* **5**, 15267; doi: 10.1038/srep15267 (2015).

## Supplementary Material

Supplementary Information

## Figures and Tables

**Table 1:** The baseline characteristics of all qualified studies.

The first author	Year	Race	Study design	Source	Criteria	AHI cutoff	Sample size		Age (years)		Males	
							Cases	Controls	Cases	Controls	Cases	Controls
Chmielewska	2013	White	Retrospective	Hospital	AASM	AHI ≥ 5	55	50	57	60	0.836	NA
Mishra (h)	2013	Asian	Retrospective	Hospital	AASM	AHI ≥ 5	216	152	46	43	0.780	0.600
Mishra (n)	2013	Asian	Retrospective	Hospital	AASM	AHI ≥ 5	179	266	46	43	0.780	0.600
Yakut	2010	Mixed	Retrospective	Hospital	Polysomnography	AHI > 5	64	37	50.37	49.97	0.828	0.703
Ogus	2010	Mixed	Retrospective	Population	Polysomnography	AHI ≥ 5	97	79	51.27	60.1	0.907	NA
Benjamin	2008	White	Prospective	Population	NA	NA	26	26	47.5	40.1	0.808	0.538
Bostrom	2007	White	Prospective	Population	Polysomnography	AHI ≥ 10	230	108	NA	NA	NA	NA
Li	2006	Asian	Retrospective	Hospital	Polysomnography		65	20	45	45	0.877	0.900
Gu	2006	Asian	Retrospective	Hospital	Polysomnography	AHI ≥ 5	124	124	5.6	4.9	0.774	0.790
Lin	2004	Mixed	Prospective	Population	Polysomnography	AHI ≥ 5	474	626	55	55	0.540	0.540
Zhang	2004	Asian	Retrospective	Hospital	Polysomnography	AHI ≥ 5	121	100	43.2	40.1	1.000	1.000
Li Y (h)	2004	Asian	Retrospective	Hospital	Polysomnography	AHI ≥ 5	30	30	44.6	45.2	0.933	0.867
Li Y (n)	2004	Asian	Retrospective	Hospital	Polysomnography	AHI ≥ 5	30	30	41.2	45.2	0.933	0.867
Li S	2001	Asian	Retrospective	Hospital	Polysomnography	AHI ≥ 5	95	50	45.37	45	0.811	0.780
Barcelo	2001	White	Retrospective	Population	Polysomnography	AHI > 20	63	32	50	49	1.000	1.000
Ping (h)	2001	Asian	Retrospective	Hospital	Polysomnography	AHI ≥ 5	41	60	60	62	0.813	0.633
Ping (n)	2001	Asian	Retrospective	Hospital	Polysomnography	AHI ≥ 5	39	60	60	62	0.813	0.633
Zhang	2000	Asian	Retrospective	Population	Polysomnography	AHI ≥ 5	61	68	54.3	52.4	0.852	0.559
Xiao (h)	1999	Asian	Retrospective	Hospital	Polysomnography	AHI ≥ 5	30	50	NA	NA	0.900	0.600
Xiao (n)	1999	Asian	Retrospective	Hospital	Polysomnography	AHI ≥ 5	20	50	NA	NA	0.900	0.600
BMI (kg/m^2^)		Smoking		Drinking		AHI		Score		Hypertension		Diabetes
Cases	Controls	Cases	Controls	Cases	Controls	Cases	Controls	Cases	Controls	Cases	Controls	Cases
33	NA	0.273	NA	NA	NA	31	NA	NA	NA	0.800	NA	0.200
32.6	27.2	0.200	0.120	0.26	0.12	36.1	0.8	10.9	6.5	1.000	1.000	0.170
32.6	27.2	0.200	0.120	0.26	0.12	36.1	0.8	10.9	6.5	0.000	0.000	0.170
30.64	28.51	NA	NA	NA	NA	NA	NA	NA	NA	NA	NA	NA
30.58	NA	NA	NA	NA	NA	24.24	NA	11.34	NA	NA	NA	0.000
38.4	NA	NA	NA	NA	NA	NA	NA	14.7	3.1	0.462	0.000	NA
NA	NA	NA	NA	NA	NA	NA	NA	NA	NA	0.700	0.000	NA
28.43	23.22	NA	NA	NA	NA	NA	NA	NA	NA	NA	0.000	NA
NA	NA	NA	NA	NA	NA	NA	NA	NA	NA	0.000	0.000	0.000
28.97	25.01	NA	NA	NA	NA	NA	NA	NA	NA	NA	0.000	NA
28.8	26.2	NA	NA	NA	NA	NA	NA	NA	NA	NA	0.000	NA
29.7	26.9	NA	NA	NA	NA	65.79	NA	NA	NA	1.000	0.000	NA
28.9	26.9	NA	NA	NA	NA	52.75	NA	NA	NA	0.000	0.000	NA
31.4	NA	0.130	NA	NA	NA	NA	NA	NA	NA	0.435	NA	NA
32.8	25.6	0.476	0.000	NA	NA	NA	NA	NA	NA	0.365	0.000	0.000
NA	NA	NA	NA	NA	NA	49	NA	NA	NA	1.000	0.000	NA
NA	NA	NA	NA	NA	NA	41	NA	NA	NA	0.000	0.000	NA
28.3	26.8	NA	NA	NA	NA	29.2	1.2	NA	NA	1.000	0.000	NA
29.5	NA	NA	NA	NA	NA	37.5	NA	NA	NA	1.000	0.000	0.000
29.5	NA	NA	NA	NA	NA	37.5	NA	NA	NA	0.000	0.000	0.000

Notes: AASM, American Academy of Sleep Medicine; NA, not available; BMI, body mass index; AHI, apnea hypopnea index; (h), OSAS patients with hypertension; (n), OSAS patients without hypertension.

**Table 2:** Overall and subgroup analysis of *ACE* gene I/D polymorphism with OSAS susceptibility under three genetic models.

Groups	Studies	I versus D		II versus DD		II plus ID versus DD	
		OR, 95% CI, P	*I*^2^	OR, 95% CI, P	*I*^2^	OR, 95% CI, P	*I*^2^
Overall	16	1.204, 0.923–1.570, 0.170	85.5%	1.337, 0.829–2.163, 0.237	81.1%	1.153, 0.827–1.607, 0.401	75.4%
Race							
Asian	9	1.443, 0.912–2.284, 0.118	90.3%	2.019, 0.867–4.701, 0.103	87.6%	1.453, 0.827–2.555, 0.194	81.5%
White	4	0.890, 0.600–1.319, 0.560	51.9%	0.812, 0.369–1.787, 0.604	52.7%	0.742, 0.446–1.233, 0.250	34.3%
Mixed	3	1.082, 0.923–1.570, 0.691	68.3%	1.120, 0.606–2.071, 0.718	49.8%	1.175, 0.608–2.270, 0.631	75.5%
Language							
English	9	1.107, 0.878–1.396, 0.389	70.5%	1.183, 0.781–1.792, 0.426	63.6%	1.039, 0.770–1.403, 0.802	58.1%
Chinese	7	1.421, 0.725–2.785, 0.306	92.1%	2.293, 0.651–8.080, 0.197	89.3%	1.618, 0.678–3.865, 0.279	85.6%
Study design							
Retrospective	13	1.334, 0.945–1.883, 0.101	86.7%	1.671, 0.892–3.132, 0.109	83.0%	1.349, 0.884–2.060, 0.165	76.5%
Prospective	3	0.854, 0.650–1.121, 0.255	48.4%	0.759, 0.469–1.230, 0.263	35.8%	0.714, 0.392–1.298, 0.269	70.2%
Control source							
Hospital-based	10	1.325, 0.865–2.031, 0.196	89.3%	1.762, 0.799–3.885, 0.160	86.3%	1.278, 0.763–2.140, 0.352	79.8%
Population-based	6	1.055, 0.779–1.429, 0.729	70.0%	1.035, 0.616–1.740, 0.895	58.7%	1.052, 0.655–1.688, 0.835	69.6%
Sample size							
<300 subjects	13	1.281, 0.861–1.905, 0.222	86.6%	1.601, 0.781–3.279, 0.199	82.1%	1.319, 0.802–2.171, 0.275	77.1%
≥300 subjects	3	1.012, 0.725–1.413, 0.945	85.5%	1.014, 0.540–1.904, 0.965	83.8%	0.906, 0.608–1.349, 0.627	75.3%
Obesity							
BMI ≥ 30 kg/m^2^	7	1.135, 0.902–1.428, 0.280	60.6%	1.261, 0.834–1.906, 0.271	53.2%	1.137, 0.870–1.485, 0.346	38.2%
BMI < 30 kg/m^2^	6	1.708, 1.047–2.785, 0.032	79.8%	2.862, 0.979–8.373, 0.055	78.1%	1.871, 0.891–3.926, 0.098	72.2%
Hypertension							
With	5	2.320, 1.587–3.390, 0.000	58.3%	3.806, 1.865–7.765, 0.000	48.2%	2.763, 1.485–5.142, 0.001	45.9%
Without	5	1.255, 0.538–2.926, 0.600	93.5%	1.815, 0.357–9.231, 0.472	92.7%	1.206, 0.515–2.824, 0.667	81.2%
Mixed	10	1.078, 0.826–1.407, 0.581	73.1%	1.047, 0.644–1.701, 0.854	63.9%	1.029, 0.704–1.502, 0.884	67.6%

Notes: OR, odds ratio; 95% CI, 95% confidence interval; BMI, body mass index.
